# Repression of CC16 by Cigarette Smoke (CS) Exposure

**DOI:** 10.1371/journal.pone.0116159

**Published:** 2015-01-30

**Authors:** Lingxiang Zhu, Peter Y. P. Di, Reen Wu, Kent E. Pinkerton, Yin Chen

**Affiliations:** 1 Department of Pharmacology and Toxicology, University of Arizona, Tucson, AZ, 85721, United States of America; 2 Department of Environmental and Occupational Health, University of Pittsburgh, Pittsburgh, PA, 15219, United States of America; 3 Center for Comparative Respiratory Biology and Medicine, University of California Davis, Davis, CA, 95616, United States of America; 4 Department of Pediatrics, University of California Davis, Davis, CA, 95616, United States of America; National Jewish Health, UNITED STATES

## Abstract

Club (Clara) Cell Secretory Protein (CCSP, or CC16) is produced mainly by non-ciliated airway epithelial cells including bronchiolar club cells and the change of its expression has been shown to associate with the progress and severity of Chronic Obstructive Pulmonary Disease (COPD). In an animal model, the lack of CC16 renders the animal susceptible to the tumorigenic effect of a major CS carcinogen. A recent population-based Tucson Epidemiological Study of Airway Obstructive Diseases (TESAOD) has indicated that the low serum CC16 concentration is closely linked with the smoke-related mortality, particularly that driven by the lung cancer. However, the study of CC16 expression in well-defined smoke exposure models has been lacking, and there is no experimental support for the potential causal link between CC16 and CS-induced pathophysiological changes in the lung. In the present study, we have found that airway CC16 expression was significantly repressed in COPD patients, in monkey CS exposure model, and in CS-induced mouse model of COPD. Additionally, the lack of CC16 exacerbated airway inflammation and alveolar loss in the mouse model. Therefore, CC16 may play an important protective role in CS-related diseases.

## Introduction

CC16 (also called CC10 or CCSP) is a homodimeric protein that was initially attributed to Club cells (formerly, Clara cells), but has since been found to be produced by other non-ciliated epithelial cells in the airway [1–5]. Besides its abundant presence in the airway lung fluids and sputum, CC16 can also be readily detected in the circulation [6–8]. The biological functions of CC16 and its affected molecular pathways have not been completely elucidated, but growing evidence indicates that CC16 has anti-inflammatory and anti-tumor functions [6–8] presumably via the inhibitions of PLA2 [9], proinflammatory prostaglandins[10,11], chemotaxis [12–16], and cytokine production [17,18]. Consistently, CC16 knockout (KO) mice had markedly enhanced inflammation and tissue remodeling in the lung when exposed to a variety of insults [19–27]. Thus, CC16 appears to play an important protective role in inflammatory lung diseases.

Recently, CC16 has raised considerable interest in smoke-related lung diseases such as Chronic Obstructive Pulmonary Disease (COPD). Although COPD is phenotypically and pathologically heterogeneous, it is now well established that chronic inflammation is at its root and generates mucus overproduction, bronchial epithelial injury, airway obstruction, and progressive deterioration in lung function. Thus, markers of systemic inflammation such as C-reactive protein (CRP) have been consistently associated with COPD morbidity and subsequent mortality [28,29]. Acute environmental exposures can cause a transient increase in CC16 level, but repeated exposures (e.g., smoking) result in chronically decreased numbers of Club cells and serum CC16 levels [30–36]. In these clinical studies, lower levels of CC16 in serum and airways have also been associated with prevalence and severity of COPD. In a recent longitudinal report from the Evaluation of COPD Longitudinally to Identify Predictive Surrogate Endpoints (ECLIPSE), serum CC16 was the only biomarker associated with a slower subsequent decline of forced expiratory volume in one second (FEV1) over 3 years [37]. This observation has been recently confirmed by another group [38]. Additionally, the recently-published long-term Tucson Epidemiological Study of Airway Obstructive Diseases (TESAOD) further indicates that low serum concentration of CC16 was strongly associated with the mortality caused by CS-related cancers, particularly lung cancer[39].

However, despite the abundant clinical data suggesting a protective role of CC16 in smoke-related lung diseases, there is no evidence to support the potential causal link between CC16 and smoke-induced pathophysiological changes. Furthermore, the present CC16 expression profile has been largely derived from the measurement of secretory CC16 in body fluids (i.e. serum, bronchoalveolar lavage, and sputum), while the studies on CC16 expression at tissue/cellular level are lacking. Thus, in the present study, we seek to fill in these gaps by examining CC16 expression in human subjects, monkey and rodent models of CS exposure, and by measuring the inflammation and alveolar destruction in mouse model of CC16 deficiency.

## Materials and Methods

### 1. Antibodies, Chemicals and Kits

CC16 antibody for human and monkey staining were purchased from BioVendor (Asheville, NC). CC16 antibody for mouse staining was purchased from Santa Cruz Biotechnology (Santa Cruz, CA). Mouse KC ELISA kit was from R&D Systems (Minneapolis, MN).

### 2. Human tissue procurement

Normal or human COPD tissues were either purchased from National Disease Research Interchange (NDRI) or obtained from the left-over autopsy samples from University of California (Davis, CA) Medical Center with previously approved protocols. All human subjects gave the consent for the tissues being used for research purposes. All tissues were de-identified and were sent to the laboratory labelled with random code without any traceable link to the individual identity. Thus, the present study does not involve human subjects as defined under HHS regulations. Normal subjects are non-smokers without diagnosed lung diseases. All moderate and severe COPD patients are current or former smokers.

### 3. Monkey model of CS exposure

All monkey tissue sections were obtained from the study as described in the previous publication [40]. Briefly, rhesus monkeys (Macaca mulatta) from the California National Primate Research Center (Davis, CA) were selected and obtained based on the approved protocol by Institutional Animal Care and Use Committee (IACUC) at the University of California Davis. Aged and diluted side-stream smoke from research cigarettes (2R4F, Tobacco Research Institute at the University of Kentucky) was produced in a smoking apparatus and was used as a surrogate for environmental smoke exposure as previously described [40]. Exposure to smoke occurred for 6 hours per day, 5 days per week at a total suspended particulate concentration of 1 mg/m3. Controls were exposed to filtered air (FA) and the rest of monkeys were exposed to smoke from 6 months of age to 13 months of age. At the end of the experiment, monkeys were sacrificed and lungs were fixed, embedded in paraffin and sectioned.

### 4. Mouse model of CS exposure

Both wild-type (WT) mice or CC16-deficient mice [41] (5–6 for each group) were either exposed to filtered air (FA) or to side-stream smoke generated by unfiltered cigarettes (1R3F research-reference cigarettes from the Tobacco Research Institute, University of Kentucky, Lexington, Kentucky) at 150 mg/m^3^, 6 hours per day, 5 days per week for 4 months following the same protocol as described by others [42]. The present animal study has been approved by Institutional Animal Care and Use Committees (IACUC) at the University of Arizona or at the University of Pittsburgh.

### 5. Measurement of tissue expression of CC16 by immunofluorescence staining

For immunofluorescence, the tissue sections were incubated with 1:100 diluted anti-CC16 antibody overnight at 4°C. Alexa 488 conjugated secondary antibody (Life Technology, Grand Island, NY) was used to obtain the fluorescence images. The images were acquired by confocal microscopy (LSM 510 meta; Carl Zeiss, Thornwood, NY).

### 6. Broncho alveolar lavage (BAL), differential cell counts, and cytokine measurement

Mice were euthanized and the trachea was then isolated via blunt dissection. Small caliber tubing was inserted and secured in the airway. Three successive volumes of PBS with 0.1% bovine serum albumin were then instilled and gently aspirated. All BAL samples collected were aliquoted and stored in three bar-coded Eppendorf tubes. The cells in the BAL were cytocentrifuged, air dried, stained with a modified Wright’s stain (Diff-Quik) and the proportion of macrophages, neutrophils, eosinophils, lymphocytes determined by counting using light microscopy. Slides were coded and be counted in a blinded manner by at least two researchers to ensure an objective evaluation. Total or differential cell numbers were presented as lavaged cells x1000. Cytokine concentrations were determined by ELISA.

### 7. Measurement of alveolar enlargement

For the qualitative assessment of the severity of emphysema, lungs were perfused and fixed by 4% paraformaldehyde at 30 cm water pressure for 1h and followed by storage of the inflation-fixed lung immersed fixatives for overnight. Morphometry was used to quantify emphysema as described previously [43–48]. Briefly, microscopic fields were chosen by a systematic random method, and emphysema was assessed in these fields with computer-aided measurements using the same software as described above. The most commonly used indicator of alveolar airspace size, the mean linear intercept (*L*_m_, in μm), was calculated. The *L*_m_ was corrected for histoprocessing shrinkage by dividing it by the group-average linear shrinkage factor.

### 8. Statistical analysis

Experimental groups were compared using a two-sided Student's t test, with significance level set as P < 0.05. When data were not distributed normally, significance was assessed with the Wilcoxon matched-pairs signed-ranks test, and P < 0.05 was considered to be significant.

## Results

### 1. CC16 was downregulated in the airways of human COPD patients

**Figure 1:**
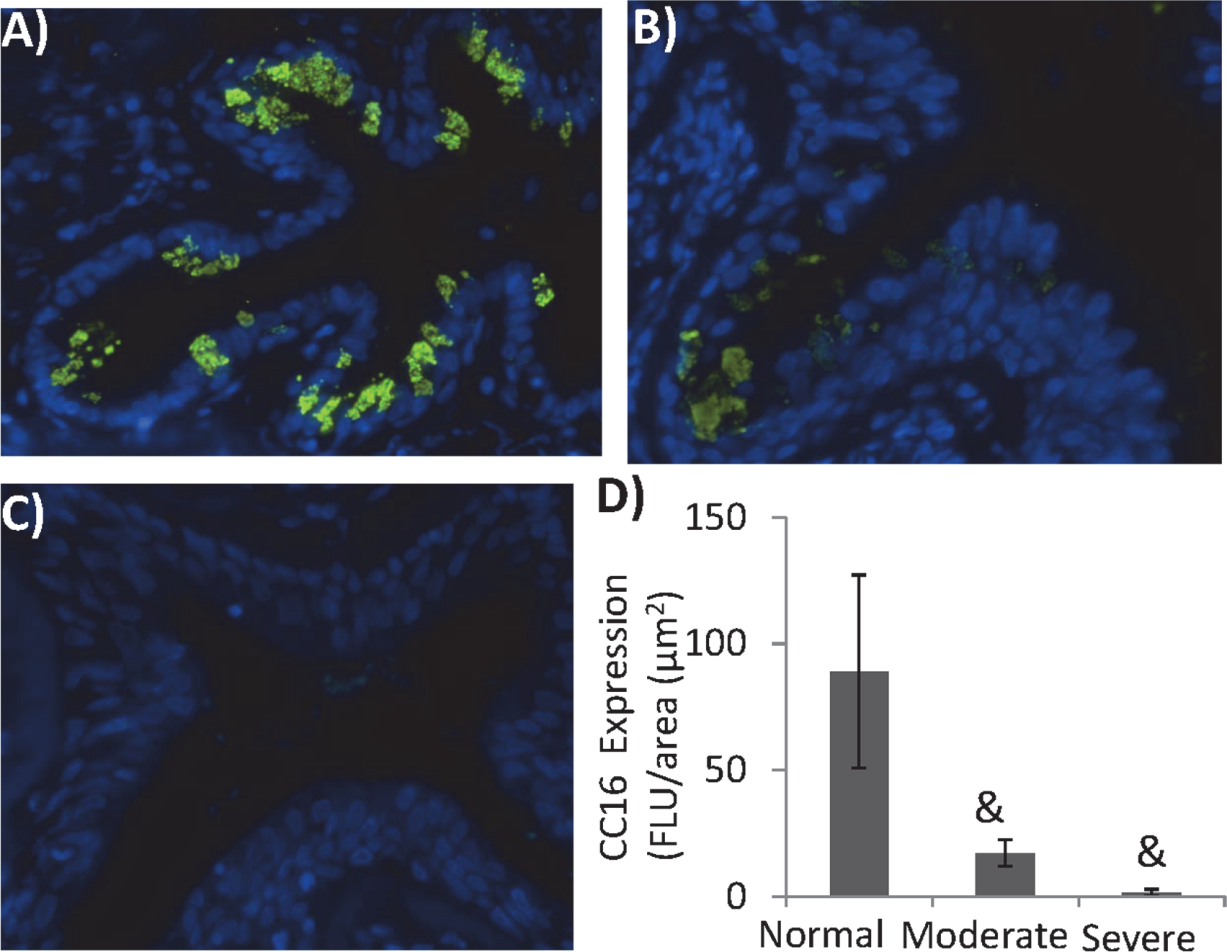
CC16 was downregulated in human COPD. Tissue sections from 5 normal subjects (A), 4 moderate (B) and 3 severe COPD patients (C) were stained with CC16 antibody (Green) and counter-stained with DAPI for nuclei (Blue). For each slide, the images were taken and quantified from at least 5 different regions of each tissue slides (D). (&: p<0.05).

Based on the previously reported epidemiological data regarding the reduction of CC16 in serum and lung BAL in smokers and COPD subjects [30–36], we further looked at the tissue-level expression of CC16 in diagnosed COPD patients. Consistently, there was a progressive decrease of epithelial CC16 expression that was correlated with the severity of COPD (Fig. 1D). Although abundant CC16 expression was observed in the normal airway (Fig. 1A), its expression was significantly reduced in the moderate COPD patients (Fig. 1B). Strikingly, CC16 almost completely disappeared from the airways of severe COPD patients (Fig. 1C). Although we could not link the level of CC16 and the severity of COPD due to the small sample size in each category, it is clear that cellular expression of CC16 was significantly downregulated in the lungs of COPD, which is consistent with the clinical studies using serum or BAL samples [30–36].

### 2. CC16 was downregulated in the airways of non-human primates exposed to CS

**Figure 2:**
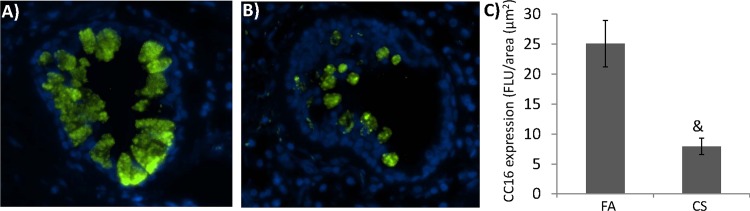
CC16 was repressed in non-human primates exposed to CS. Rhesus monkey was exposed to CS as described in Material and Method Section. Tissue sections from A) 3 filtered air (FA)- and B) 6 CS-exposed monkeys were stained with CC16 antibody (Green) and counter-stained with DAPI for nuclei (Blue). For each slide, the images were taken and quantified from at least 5 different regions of each tissue slides (C). (&: p<0.05).

Because smoke is one of key factors of COPD pathogenesis and CC16 expression has been shown to be repressed in smokers, we tried to establish a causal link between CC16 expression and CS exposure. We examined non-human primate model of CS exposure. 7-month CS exposure significantly reduced CC16 expression in the lung (Fig. 2B-C) as compared to the filtered air (FA)-exposed controls (Fig. 2A, C).

### 3. The lack of CC16 exacerbated CS-induced inflammation and lung destruction

**Figure 3:**
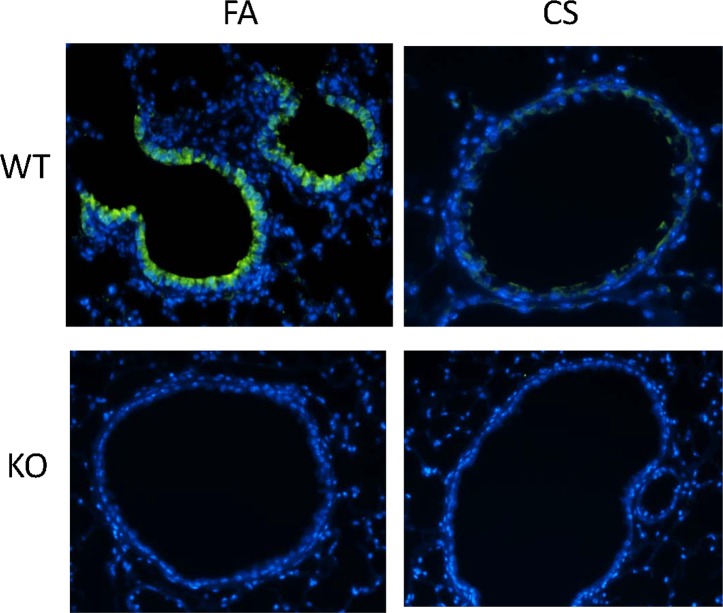
CC16 was downregulated in WT mouse when exposed to CS. Both WT and CC16 KO mice were exposed to CS. CC16 staining (Green) was performed on lung tissues from WT or KO mice exposed to FA or CS.

**Figure 4:**
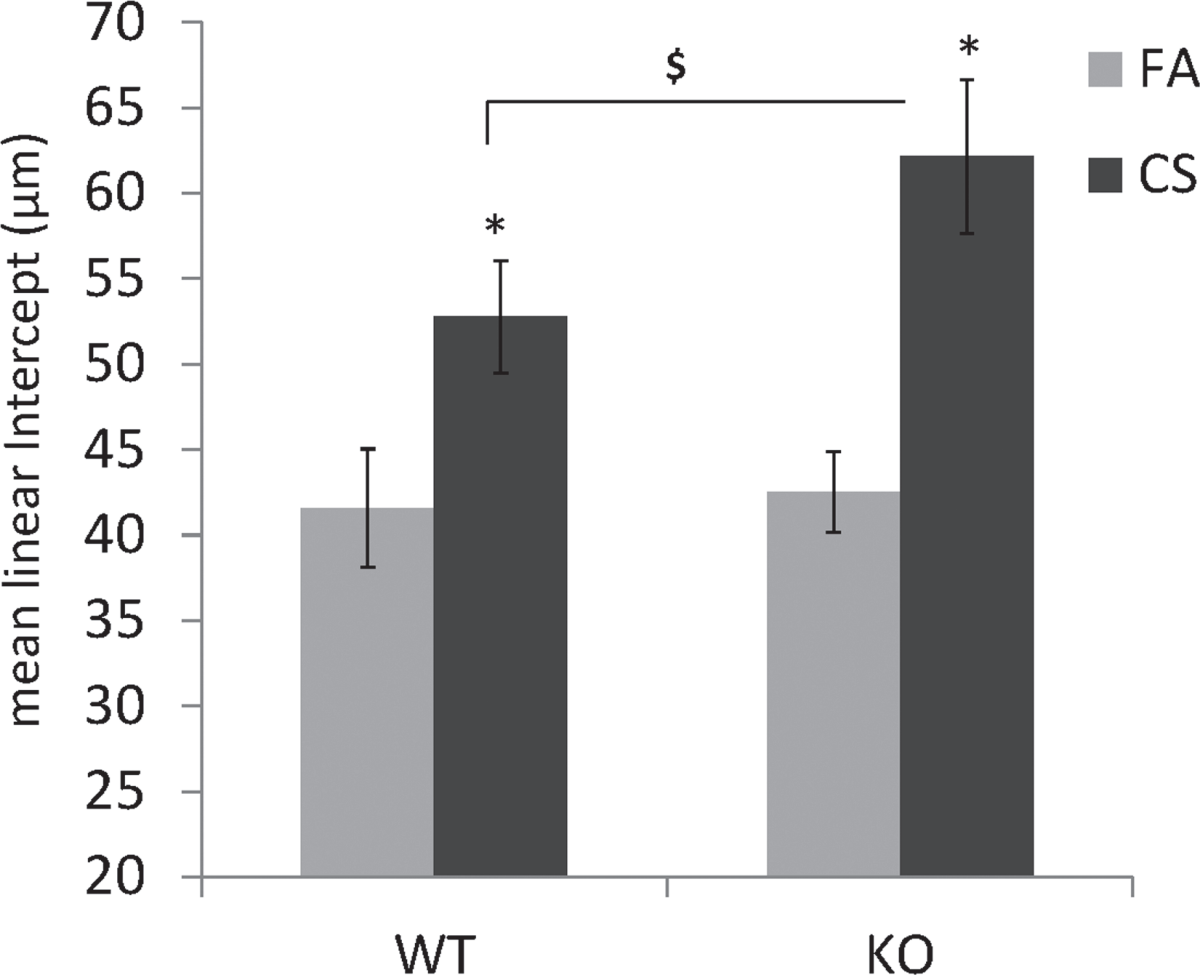
Enlargement of Alveolar Space (represented by the mean linear intercept, μm) *: p<0.05, CS vs. FA. $: p< 0.05, KO+CS vs. WT +CS.

CC16 has a broad range of anti-inflammatory functions [9–18]. In the lung, CC16 KO mice had markedly enhanced inflammation and tissue remodeling when exposed to a variety of insults [19–25]. However, the effect of CC16 deficiency has not been established in the models of smoke exposure or smoke-induced COPD. Thus, we examined CS-induced pathophysiological changes in both WT and CC16 KO mice. As shown in Fig. 3, WT mice had similar CC16 reduction in their lungs when exposed to CS as the primates (Fig. 2), suggesting CS-repressed CC16 expression may be an evolutionarily conserved response. As expected, CS significantly elevated total inflammatory cell counts in BAL from WT mice, and most of these cells were macrophages and neutrophils (Table 1). As compared to WT mice, KO mice had much worse inflammatory responses as demonstrated by much higher total cell counts, differential cell counts and proinflammatory cytokine (KC) production (Table 1). Thus, the lack of CC16 exacerbated CS-induced inflammatory responses. Furthermore, we measured alveolar regions of these mice, and found out that the mice exposed to CS had significantly enlarged alveolar airspace (measured by the mean linear intercept) as compared to the mice exposed to filtered air (FA) (Fig. 4). Additionally, the alveolar airspace of KO mice was even more enlarged than WT mice, indicating more alveolar loss induced by CS exposure. Taken together, the lack of CC16 severely exacerbates CS-induced inflammation and alveolar pathology, suggesting that CC16 may play an indispensable protective role in smoke-related diseases such as COPD.

**Table 1:** Exacerbated inflammation in CC16 deficient mice.

		Total Cells	Macrophage	Neutrophil	Lymphocyte	KC (pg/ml)
WT	FA	260±88	253±88	4±2	3±2	32.6±16.2
	CS	2259±689*	1407±465*	831±224*	21±8 *	346.5±72.5 *
KO	FA	291±62	279±61	7±1	4±1	28.7±10.6
	CS	3983±935* ^,^ ^$^	2461±630 * ^,^ ^$^	1482±365* ^,^ ^$^	39±16 *	778.7±152.2* ^,^ ^$^

Differential cell counts (x1000) and cytokine concentration were measured in BAL.

*: p<0.05, CS vs. FA.

^$^: p<0.05, KO+CS vs. WT +CS.

## Discussion

CC16 has emerged as an important clinical biomarker in various inflammatory lung diseases including COPD [30–36]. The significant repression of CC16 in the serum of COPD patients and its inverse relationship with the decrease of lung function raise considerable interest of using this protein as a peripheral biomarker for the COPD progression [37,38]. In serum, CC16 concentration is determined by both the production mainly form the lung and the clearance by kidney [49] due to its small size. In the lung, two major processes appear to determine the intrapulmonary pool of CC16. First, CC16 is produced from club cells and other non-ciliated airway epithelial cells [1–5]. Second, the leakage or active transportation moves CC16 from lung compartment to the circulation [7]. In chronic diseases such as COPD, the epithelial barrier function is generally compromised which leads to high lung permeability[50]. Thus, when both systems (i.e. pulmonary and peripheral) are combined, decreased serum CC16 level is most likely caused by the reduced CC16 production and/or the increase of renal function. Interestingly, some studies [31,33] indicates that CC16 levels in BAL were also decreased in these patients, suggesting that a significant part of serum CC16 decrease was perhaps the result of reduced intrapulmonary CC16 pool. Consistent with these reports, our data indicates that the tissue CC16 expression was markedly decreased in the airway epithelia of human COPD and of the CS-exposed animal models of monkey and mouse. The significant reduced CC16 expression in airway epithelium may also be caused by the loss of a specific cell type that expresses CC16. In the past, club cells have been thought to be the main source of lung CC16 production. However, CC16 expression has later been found to occur in many other non-ciliated airway epithelial cells that are morphologically different from club cells [1]. Thus, the most likely explanation is that CS exposure may repress CC16 expression in many different cell types, rather than merely eliminate the club cells. Recently, CC16 has been found to be one of the top hypermethylated genes in airway epithelial cells from smokers as compared with nonsmokers [51], which supports the idea that chronic CS-exposure shuts down CC16 expression possibly via an epigenetic mechanism.

Although the epidemiological data convincingly link the expression of CC16 with the severity of COPD, the evidence supporting the potential causality between the two has been lacking. Smoking or environmental smoke exposure has been considered as one of the dominant factors for COPD. When exposed to smoke-derived carcinogen, the mice lacking CC16 had higher incidence of airway epithelial hyperplasia, lung adenoma and elevated level of MAP Kinase activation [23] supporting the role of CC16 in chemoprevention. Because MAP kinase pathway is critically involved in smoke induced lung inflammation [52], this finding [23] also implies that CC16 may play a role in airway inflammation. Indeed, we have demonstrated in the present study that the lack of CC16 substantially exacerbated CS-induced airway inflammation and alveolar loss. Our finding is also in line with the previous reports about markedly heightened inflammation and airway remodeling in a variety of other lung disease models [19–27] when CC16 was knocked out. Surprisingly, one recent study has found no significant difference between WT and CC16 KO mice after being exposed to CS [38]. The cause of this discrepancy between the two studies is currently unknown. It may have to do with the status of KO mouse, the CS exposure protocol or the assays. Nonetheless, we have provided the positive evidence supporting the anti-inflammatory role of CC16 in smoke-related airway disease model.

CC16 protein and its truncated C-terminal peptide have been proposed to treat a variety of inflammatory lung diseases (i.e., neonatal respiratory distress syndrome (nRDS), acute respiratory distress syndrome (ARDS), asthma and CF) [53]. Recombinant human CC16 (rhCC16) has been evaluated in clinical trials for treating nRDS [54,55] and allergic rhinitis [56] with some success. Interestingly, a single intratracheal dose (5mg/kg) of rhCC16 resulted in a significant reduction of neutrophil and total cell counts and lung protein concentration in nRDS, confirming the anti-inflammatory function of CC16 in human subjects [54,55]. In the literatures, the function of CC16 has been attributed to its inhibition of PLA2 activity[9], pro-inflammatory prostaglandins [10,11][10,11], chemotaxis [12–16], and cytokine production [17,18]. However, whether or not any of these molecular pathways is responsible for the protective function of CC16 in smoke-related diseases including COPD has not been proved. Our results as demonstrated here will provide important rationale and the useful models for the future study of CC16 function. It is our opinion that CC16 is not only an important peripheral disease marker, but also a valuable druggable target. Therefore, further understanding of the biological function of CC16 in the context of COPD and other CS-associated diseases is warranted and the progress at this front is vital to our endeavor to develop therapeutics for the treatment of these chronic airway diseases.

.

## Supporting Information

S1 ARRIVE Checklist. (DOCX)
